# OATargets: a knowledge base of genes associated with osteoarthritis joint damage in animals

**DOI:** 10.1136/annrheumdis-2020-218344

**Published:** 2020-10-19

**Authors:** Jamie Soul, Matthew J Barter, Christopher B Little, David A Young

**Affiliations:** 1 Skeletal Research Group, Biosciences Institute, Newcastle University, Newcastle upon Tyne, Tyne and Wear, UK; 2 Raymond Purves Bone and Joint Research Laboratories, Kolling Institute, The University of Sydney, St Leonards, New South Wales, Australia

**Keywords:** osteoarthritis, chondrocytes, knee

## Abstract

**Objectives:**

To collate the genes experimentally modulated in animal models of osteoarthritis (OA) and compare these data with OA transcriptomics data to identify potential therapeutic targets.

**Methods:**

PubMed searches were conducted to identify publications describing gene modulations in animal models. Analysed gene expression data were retrieved from the SkeletalVis database of analysed skeletal microarray and RNA-Seq expression data. A network diffusion approach was used to predict new genes associated with OA joint damage.

**Results:**

A total of 459 genes were identified as having been modulated in animal models of OA, with ageing and post-traumatic (surgical) models the most prominent. Ninety-eight of the 143 genes (69%) genetically modulated more than once had a consistent effect on OA joint damage severity. Several discrepancies between different studies were identified, providing lessons on interpretation of these data. We used the data collected along with OA gene expression data to expand existing annotations and prioritise the most promising therapeutic targets, which we validated using the latest reported associations. We constructed an online database OATargets to allow researchers to explore the collated data and integrate it with existing OA and skeletal gene expression data.

**Conclusions:**

We present a comprehensive survey and online resource for understanding gene regulation of animal model OA pathogenesis.

**Key messages**

**What is already known about this subject?**

- Animal models are commonly used as preclinical discovery tools to study osteoarthritis (OA).
- Genes are often modulated in these animal models to understand pathogenic signalling or recapitulate a disease modifying treatment scenario.

**What does this study add?**

- A knowledge base of all genes modulated in animal models of OA and integration with all publicly available OA transcriptomics data.
- Prioritisation approach for expanding known functional OA genes—validated using the latest reported research findings.

**How might this impact on clinical practice or future developments?**

- The knowledge base provides a roadmap to pinpoint druggable functional OA candidates for future therapies.

## Introduction

Animal models have been used widely in the study of osteoarthritis (OA) as preclinical discovery tools to identify key molecular mechanisms contributing to OA pathophysiology⁠⁠.^1 2^ Animal models are a powerful research tool allowing the controlled study of the earliest time points of OA initiation through disease progression, assessing joint-wide pathology and omics analysis which is not possible in human tissues⁠⁠.^3 4^ There are a lack of validated *in vitro* models for OA with these models primarily consisting of cell or tissue-based systems, usually from a single-joint tissue, with supraphysiological levels of cytokines under glucose rich and normoxic conditions, that have uncertain relevance to the *in vivo* disease⁠.^5^ The use of animal models overcomes some of the limitations of human *ex vivo* OA culture models, potentially allowing more translatable research not only with modelling of pathology of the whole joint but also clinically-relevant pain outcomes⁠.^6^

Previous publications have reviewed the range of OA animal models with regard to species, and mode of OA initiation, and described their relative advantages and limitations.^1 7–9^ These animal models fall into broad categories of: (1) post-traumatic OA through surgical and mechanical (injurious load, excessive exercise) induction, with varying severity depending on the injury target (eg, meniscus, cruciate ligament, intra-articular fracture), (2) mouse strains with increased genetic susceptibility (eg, *Col9a1* or *Col2a1* mutant, STR/ort mice), (3) metabolic/obesity induced by high-fat diet, (4) hormonal induced by ovariectomy, (5) chemically induced (eg, monosodium iodoacetate, collagenase) and (6) spontaneous/age-associated OA.^6 10^ These animal models are often genetically tractable allowing knockout, transgenic overexpression or knock-in mutation of genes, to investigate and define the key regulators of pathogenic joint signalling. In addition, these models have been used with interventions in the form of treatment with drugs, antibodies, transient gene/protein overexpression or knock-down which may better recapitulate the effect of gene modulation in a disease modifying treatment scenario.

There is no up-to-date database describing what genes have been manipulated in OA animal models and the effect on the resulting OA phenotypes.^2^ The results of these interventions in animal models are primarily available in fragmented publications, hindering efforts to learn from previous work. This study aimed to bring together this knowledge to gain an overview of the use of genetic manipulation in animal model OA research to investigate OA pathophysiology. We compare the OA-associated genes with OA transcriptomic data, prioritise yet unstudied genes, and for the first time provide an updatable resource for rapidly exploring evidence for candidate gene involvement in OA and target tractability.

## Materials and methods

### Literature search

A systematic search for publications describing animal models of OA was performed in PubMed, searching for English-language articles published between 1 January 2000 and 29 July 2020 using the following terms in combination with ‘osteoarthritis’; ‘mouse’, ‘mice’, ‘rat’, ‘*in vivo*’, ‘animal model’. Papers were curated to retain reports of genetic (knockout/in, overexpression) or exogenous (virus, protein, antibody, drugs with defined structure and targets) interventions and the resulting effect (or lack of effect) on incidence/severity of OA in animal models including any one of cartilage degradation, proteoglycan loss, subchondral bone remodelling/sclerosis, osteophytes and synovitis, but excluding solely pain. Reports with both increased and decreased observed severity in different tissue types were recorded as having a mixed effect. Models of inflammatory arthritis such as interleukin-1b (IL1B), tumour necrosis factor α (TNF), collagen-induced or antibody-induced arthritis were excluded.

### Labelling the effects of gene modulations on OA severity

The types of gene modulation (increase or decrease in gene activity) and the observed effects on OA phenotypes were used to label the 459 unique modulated genes as ‘protective’, ‘detrimental’ or ‘no effect’ for each individual experiment. For both the gene expression comparison and network expansion, non-protein coding genes were removed and the individual experiment inferred effects for each of the 425 protein-coding genes were combined. Genes were labelled ‘ambiguous’ if there was disagreement in direction of effect between experiments (ie, both protective and detrimental effects reported). Observations of no effect were considered superseded by any observation of a significant effect (positive or detrimental) on OA phenotypes for that modulated gene.

### Gene expression analysis

All available animal model and human OA transcriptomic datasets (cartilage, bone, synovium, whole joint) were downloaded from SkeletalVis (http://skeletalvis.ncl.ac.uk/skeletal) on 9 June 2020.^11^ Differentially expressed genes (absolute ≥1.5 fold change and adjusted p value ≤0.05) were used to find enriched Reactome pathways with goseq (adjusted p value ≤0.05).^12 13^⁠ Gene identifiers were mapped to human gene symbols via Ensembl orthologs. miRNA entries were removed for the comparison as small RNA expression datasets are not included in SkeletalVis. The Fishers exact test with Benjamini-Hochberg multiple testing correction was used to test gene set overlaps. χ^2^ tests with Benjamini-Hochberg correction were used to test the proportions of protective and detrimental genes or surgical and spontaneous model genes in the overlaps.

### Network expansion of OA-associated genes

A network diffusion algorithm was used to rank genes based on network proximity to OA genes with an effect on OA severity, and repeated cross validation was used to test the predictive performance of this approach (online supplemental methods).^14^ Newly reported animal model OA associations from the 2020 Osteoarthritis Research Society International (OARSI) conference abstracts were used to test prioritisation performance on new data. The Wilcox test was used to test the expected and the observed gene ranks. OA genome-wide association study (GWAS) signal variants were retrieved from a recent review⁠.^15^ Target drug tractability information was obtained from the OpenTargets platform.^16^

10.1136/annrheumdis-2020-218344.supp1 Supplementary data

### Data availability

Data and code to reproduce the analysis are available at www.github.com/soulj/OATargets.

## Results

### Summary of genes modulated in animal models of OA

**Figure 1:**
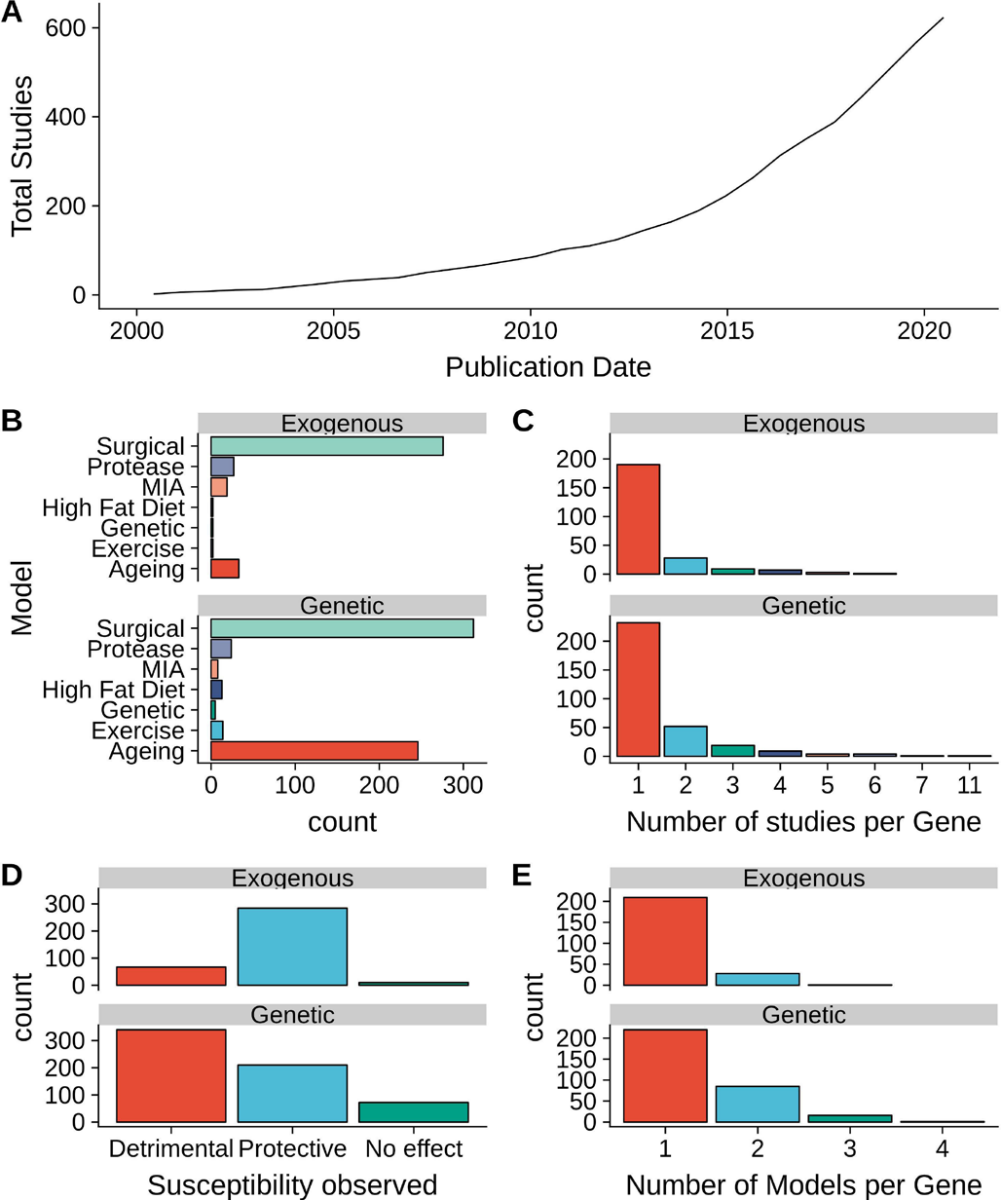
Summary of studies examining susceptibility to osteoarthritis (OA) after gene modulation. (A) Cumulative number of studies by published date. (B) Number of individual gene modulations per type of OA animal model. (C) Number of individual studies reporting each modulated gene. (D) Total number of observations by susceptibility observed. (E) Number of OA animal models used per gene studied. MIA, monosodium iodoacetate.

Search and curation of the literature for reports of OA susceptibility or progression in animal models after gene modulation identified 623 publications with 459 unique modulated genes (termed "OA genes" henceforth) with an increase in the rate of publications from 2000 to 2020 (figure 1A). Observations from these studies were grouped into genetic modulations (eg, overexpression, knockout, knockin) or exogenous modulations (eg, transient knock-down, drug treatment). In total, 415 publications reported 622 observations of genetic modulations of 322 unique genes, and 266 publications described 361 exogenous modulations of 238 unique genes (online supplemental table 1). Post-traumatic (surgical) and spontaneous/ageing models were found to be the most prevalent models of OA in genetic interventions, while the exogenous modulations were primarily performed in surgical models (figure 1B). Most of the studied genes, in both genetic and exogenous interventions, were reported in a single study and in a single type of OA model (figure 1C, E). The majority of genetic manipulation studies reported detrimental outcomes while exogenous interventions primarily reported improvement of OA phenotypes (figure 1D). All genetic studies identified were performed in mice, while greater diversity of species was used in exogenous modulations, including studies in rat and rabbit models.

10.1136/annrheumdis-2020-218344.supp2 Supplementary data

To facilitate assessment of consistency between experiments, the types of gene modulation (increase or decrease in gene activity) and the observed effects on OA phenotypes were used to label the modulated genes as protective, detrimental or no effect OA genes in each experiment, akin to the idea of an oncogene versus a tumour suppressor in cancer. For instance, a protective label was inferred if an increase in OA severity was observed on inhibition of a gene, while inhibition of a detrimental gene would attenuate OA progression. Using this approach, 98/143 genes (69%) genetically modulated more than once had a consistent inferred effect on OA (online supplemental table 1). Similarly, 61/74 (82%) genes studied multiple times in the exogenous model had the same inferred effect. Examples of genes with inconsistent results are shown in table 1. A total of 101 genes were studied through both genetic and exogenous approaches, of these 71 (70%) had consistent results, although several of these findings are reported from within the same publication or research group. Interestingly, 68/82 (83%) genes with unambiguous effects within the same OA model were consistent in their inferred effect between spontaneous and surgical models. Examples of genes confirmed in multiple models both through genetic and pharmacological means in separate studies are shown in table 2.

**Table 1:** Examples of gene modulations in osteoarthritis (OA) models with discrepancies

Human gene	PMID	Intervention	Effect on protein product	OA model	Observed effect	Inferred gene effect	Specificity	Species
Mixed effects between models and tissue specificity								
EZH2	30327434	Knockout	Removal	Spontaneous	No effect	No effect	Cartilage	Mouse
	31910305	Knockout	Removal	Surgical − MM	Detrimental	Protective	Cartilage inducible	Mouse
	27539752	EPZ005687	Inhibition	Surgical − ACLT	Protective	Detrimental	Articular cavity	Mouse
Opposite effects between models								
MINK1	31647983	Knockout	Removal	Spontaneous	Protective	Detrimental	Global	Mouse
		Knockout	Removal	Surgical − DMM	Detrimental	Protective	Global	Mouse
Detrimental effects with any gene modulation								
TTR	28941045	Knockout	Removal	Surgical − DMM	Detrimental	Detrimental	Global	Mouse
		Knockout	Removal	Spontaneous	Detrimental	Detrimental	Global	Mouse
		Overexpression	Overexpression	Surgical − DMM	Detrimental	Protective	Global	Mouse
Effect observed in only one type of model								
CD9	27784871	Knockout	Removal	Surgical - MML+ MCL	No effect	No effect	Global	Mouse
		Knockout	Removal	Spontaneous	Protective	Detrimental	Global	Mouse
TLR4	31044181	Knockout	Removal	High fat diet	Protective	Detrimental	Global	Mouse
	26245312	Knockout	Removal	Surgical − DMM	No effect	No effect	Global	Mouse
	24703622	Knockout	Removal	Surgical − DMM + MM	No effect	No effect	Global	Mouse
Potential effect of direction of gene modulation, model or tissue specificity								
RHEB	29991473	Knockout	Removal	Collagenase	Protective	Detrimental	Macrophage	Mouse
	31229684	Overexpression	Overexpression	Surgical − DMM	Protective	Protective	Articular cartilage	Mouse

Examples of gene perturbations in animal models of OA with disagreements in the inferred gene effect are shown.

ACLT, anterior cruciate ligament transection; DMM, destabilisation of the medial meniscus; MCL, medial collateral ligament; MM, medial meniscectomy; MML, medial meniscotibial ligament.

**Table 2:** Examples of gene modulations in osteoarthritis (OA) models with both genetic and exogenous evidence

Human gene	PMID	Intervention	Effect on protein product	OA model	Observed effect	Inferred gene effect	Specificity	Species
SIRT1	23723318	Knockout	Removal	Surgical − DMM +MM	Detrimental	Protective	Cartilage	Mouse
	32665267	Knockout	Removal	Surgical − DMM	Detrimental	Protective	Cartilage	Mouse
	32499111	Knockout	Removal	Surgical − DMM	Detrimental	Protective	Cartilage Inducible	Mouse
	32499111	Knockout	Removal	Spontaneous	Detrimental	Protective	Cartilage Inducible	Mouse
	23723318	Knockout	Removal	Spontaneous	Detrimental	Protective	Cartilage	Mouse
	23587642	Knockout	Removal	Spontaneous	Detrimental	Protective	Global	Mouse
	23124828	Mutation	Inhibition	Spontaneous	Detrimental	Protective	Global	Mouse
	22258484	Haploinsufficiency	Deficiency	Spontaneous	Detrimental	Protective	Global	Mouse
	29922443	SRT1720	Activation	Surgical − DMM	Protective	Protective	Systemic	Mouse
	31989845	SRT2104	Activation	Surgical − DMM	Protective	Protective	Articular cavity	Mouse
FYN	29555825	Knockout	Removal	Spontaneous	Protective	Detrimental	Global	Mouse
	29555825	Knockout	Removal	Surgical − DMM	Protective	Detrimental	Global	Mouse
	31534047	Knockout	Removal	Surgical − DMM	Protective	Detrimental	Global	Mouse
	29555825	PP1	Inhibition	Surgical − DMM	Protective	Detrimental	Systemic	Mouse
	29555825	AZD0530	Inhibition	Surgical − DMM	Protective	Detrimental	Systemic	Mouse
TNFRSF11B	30623241	Knockout	Removal	Surgical – TMJ	Detrimental	Protective	Global	Mouse
	27541035	Knockout	Removal	Spontaneous	Detrimental	Protective	Global	Mouse
	26018435	Knockout	Removal	Spontaneous	Detrimental	Protective	Global	Mouse
	17907189	Haploinsufficiency	Deficiency	Spontaneous	Detrimental	Protective	Global	Mouse
	17907189	Haploinsufficiency	Deficiency	Surgical − DMM	Detrimental	Protective	Global	Mouse
	17907189	Protein	Increase	Surgical − DMM	Protective	Protective	Articular cavity	Mouse
	18668550	Protein	Increase	Surgical − DMM + MM	Protective	Protective	Articular cavity	Mouse
	23723320	Protein	Increase	MIA	Protective	Protective	Systemic	Rat
ADAMTS5	21337391	Knockout	Removal	Surgical − DMM	Protective	Detrimental	Global	Mouse
	21337391	Knockout	Removal	Treadmill + TGFB	Protective	Detrimental	Global	Mouse
	19010693	Knockout	Removal	Surgical − DMM	Protective	Detrimental	Global	Mouse
	17968948	Knockout	Removal	Surgical − DMM	Protective	Detrimental	Global	Mouse
	15800624	Knockout	Removal	Surgical − DMM	Protective	Detrimental	Global	Mouse
	23954517	Antibody	Inhibition	STR/ort	Protective	Detrimental	Articular cavity	Mouse
	26410555	Antibody	Inhibition	Surgical − DMM	Protective	Detrimental	Systemic	Mouse
	28120109	siRNA	Knockdown	Surgical − DMM	Protective	Detrimental	Articular cavity	Mouse

Examples of gene perturbations in animal models of OA with data from both genetic and exogenous interventions are shown.

DMM, destabilisation of the medial meniscus; MIA, monosodium Iodoacetate; MM, medial meniscectomy; TMJ, temporomandibular joint hyperocclusion.

### Integration with OA transcriptomics data

To investigate the regulation of these 459 unique genes modulated in animal models (OA genes), 57 expression profiles identifying differential gene expression in human OA and animal model OA were examined (online supplemental table 2)^⁠^.^3 17–39^ Enrichment analysis showed statistically significant overlap between the 425 protein coding OA genes and the sets of differentially expressed genes, regardless of species and OA model (online supplemental figure 1). A total of 70% (298/425) and 80% (340/425) of the protein-coding OA genes were found to be differentially expressed in at least one human OA and animal model expression dataset, respectively. However, this observation is confounded by the use of existing knowledge of gene differential regulation to choose which genes to modulate in animal models.

10.1136/annrheumdis-2020-218344.supp3 Supplementary data

10.1136/annrheumdis-2020-218344.supp4 Supplementary data

**Figure 2:**
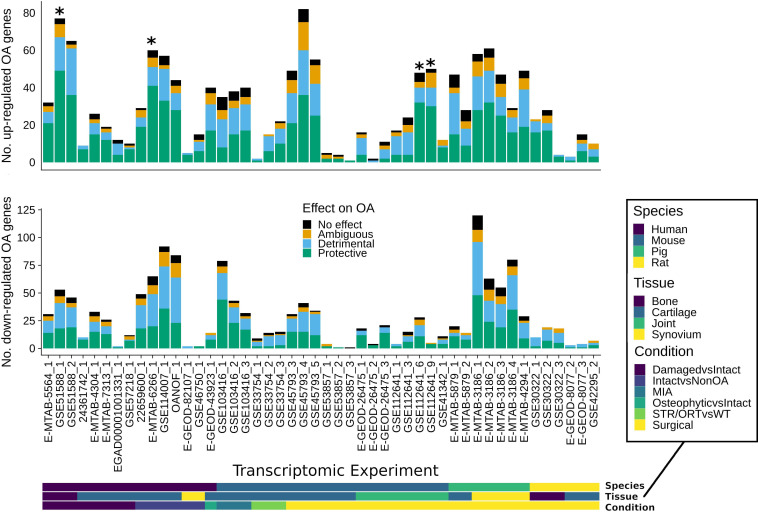
Differential expression of the osteoarthritis (OA) genes. The overlaps of upregulated and downregulated animal model or human OA differentially expressed genes with protective or detrimental OA genes are shown. Stars indicate statistically significant (Benjamini-Hochberg p value ≤0.05) differences in the proportions of differentially expressed protective and detrimental OA genes. The species, condition and tissue of the gene expression studies are indicated in the bottom bars. MIA, monosodium iodoacetate.

The individual observations for each OA gene were combined to label each gene with a consensus inferred effect (see methods). Both protective and detrimental OA genes were found to be differentially expressed in datasets across species and tissues in generally similar proportions, suggesting the direction of OA expression changes is not typically indicative of protective or detrimental effects of OA genes on modulation in induced OA (figure 2). Genes with solely no effect observations were also often differentially expressed, suggesting disease-associated regulation is not necessarily indicative of functional effects on modulation in induced OA. Interestingly, in the genes upregulated in human intact OA cartilage compared with non-OA cartilage, there was a statistically significant proportion of protective compared with detrimental OA genes (online supplemental table 2). These protective OA genes include extracellular matrix genes and growth factors that are upregulated in the human intact OA versus non-OA cartilage suggesting a protective anabolic response in the intact OA cartilage. These results suggest that the curated OA genes from mixed animal models are consistently differentially regulated across a range of tissues and species.

### Network expansion of OA-associated genes

The collated data allow a genome-wide view of the pathways that when altered enhance or protect against induced OA. Pathway analysis identified 128 pathways significantly enriched in the OA genes (online supplemental table 3). Of the human Reactome pathways, 44% (961/2203) are covered by at least one OA gene, suggesting a large coverage of known signalling pathways (online supplemental table 3).

10.1136/annrheumdis-2020-218344.supp5 Supplementary data

**Figure 3:**
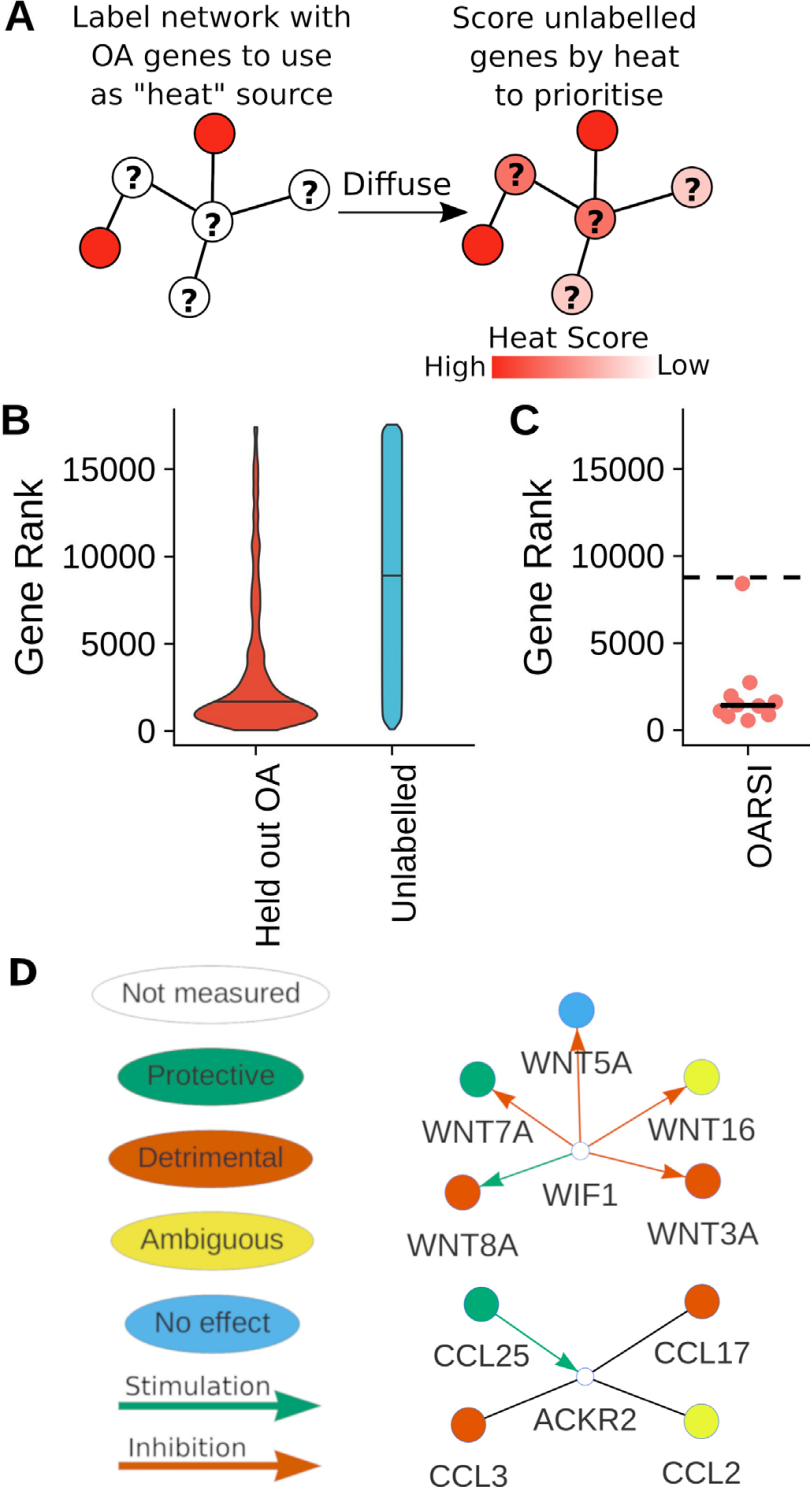
Expansion of known osteoarthritis (OA) genes. (A) Schematic of network diffusion algorithm used to expand known OA genes. (B) Violin plots demonstrating the ability to recover held-out known OA genes on the basis of network topology. Network diffusion-based ranks of held out known OA genes and unlabelled genes from 100 repeats of fivefold cross validation. (C) Network ranks of the latest reported OA genes from conference abstracts. Expected mean random rank showed by dashed line. (D) Example networks of a highly ranked genes (white), showing interactions to OA genes. The inferred effect of the OA genes and known regulatory interactions are indicated.

Analysis of associated genes from human diseases has suggested the presence of protein–protein interaction (PPI) network disease modules where groups of disease-related genes in the same signalling pathways occur⁠.^40^ To examine if OA genes can be predicted based on pathways, we tested the ability of a network diffusion algorithm to successfully recover hidden (held-out) OA genes (figure 3A). Across 100 random samples of OA genes with an effect on OA severity (ie, not labelled as no effect), the median rank of the held-out OA genes was 1575/17557 compared with 8965 for unlabelled (not known to be associated) genes, suggesting the held-out OA genes can be successfully recovered (figure 3B). This network approach allows identification of highly ranked unlabelled genes which are potential OA genes, therefore enabling expansion of OA signalling pathways.

To further prioritise genes, all OA genes with an effect on OA severity were input into the diffusion algorithm. This approach significantly prioritised the separate validation dataset of the latest potential associations from newly published conference abstracts (p value 0.001953) (figure 3C, online supplemental table 4). Interestingly, several yet unstudied genes nearest (upstream or downstream) to OA GWAS variants were also highly ranked, allowing prioritisation of these candidates. These resulting predictions were combined with differential expression in human OA expression datasets to provide orthogonal evidence of relevance to human OA (table 3). For example, the highly ranked *ACKR2* is differentially expressed in multiple human OA datasets and is a receptor for several chemokines known to affect OA in animal models, making it a potential candidate for future study and illustrating how relevant pathways can be systematically expanded using previously studied genes and available expression data (figure 3D).

10.1136/annrheumdis-2020-218344.supp6 Supplementary data

**Table 3:** Potential regulators of osteoarthritis (OA) severity

Gene name	No of human studies differentially expressed	No. interactions with OA genes	Rank
ACKR2	4	4	241
SAT2	3	5	263
NOG	4	4	311
FBLN2	4	4	356
FZD9	5	4	361
MMP14	4	4	362
WIF1	3	5	363
FZD8	6	6	381
ITGA11	6	4	387
CD36	3	11	395

The top predicted regulators using network-based expansion of the OA genes are shown. Regulators were filtered to be differentially expressed in at least three human OA expression datasets, to have at least four interactions with known OA genes and to exclude known OA genes. The rank of the network-based score is shown out of 17 557 total genes in the network.

### Knowledgebase of OA modulations in animal models

**Figure 4:**
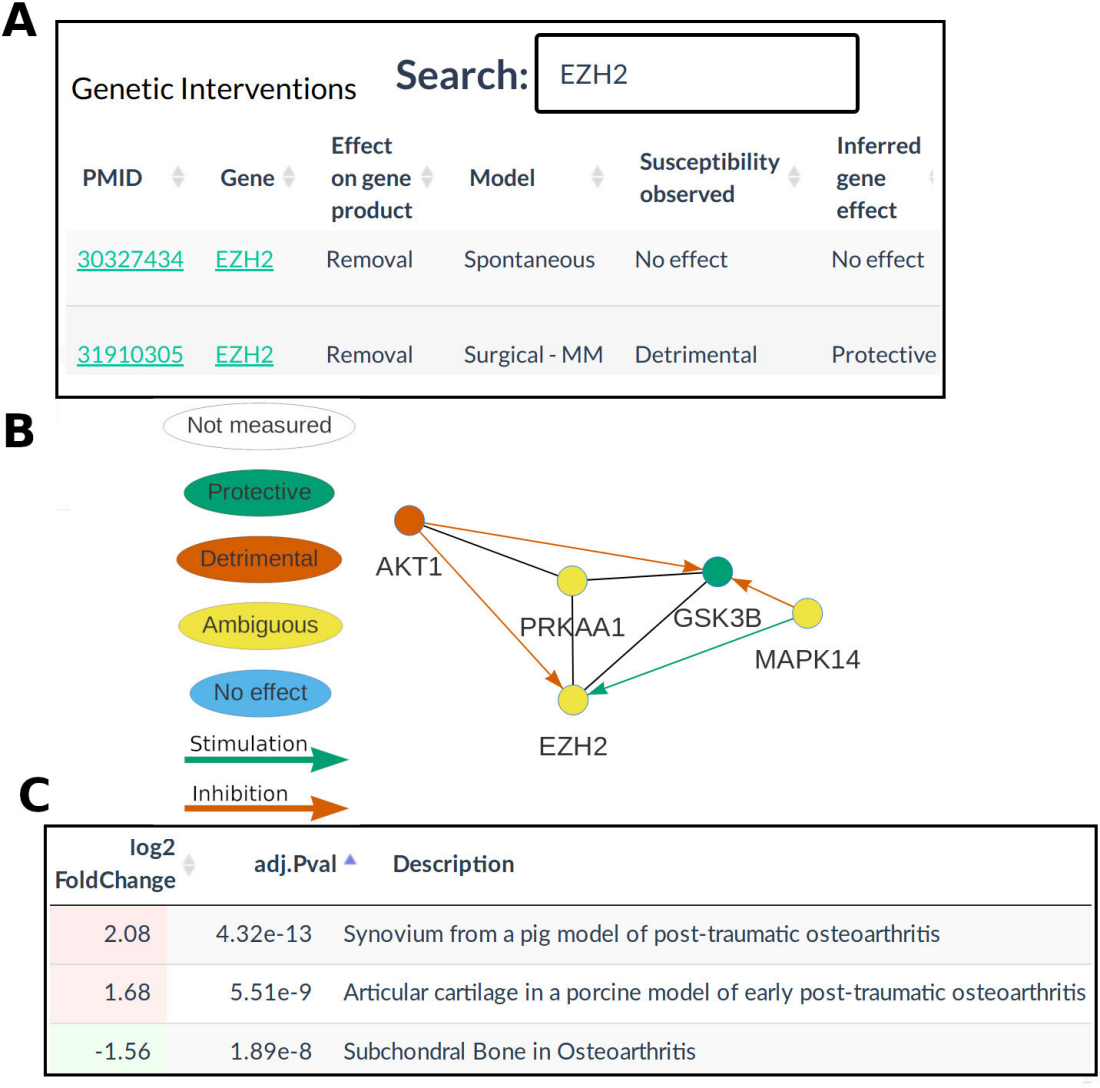
Database of osteoarthritis (OA) models and targets. Analysis of genes modulated in OA animal models (OA genes) with the OATargets database. (A) The database provides searchable tables of curated data. (B) interactive protein interaction networks with (C) links to an existing gene expression database. MM, medial meniscectomy.

To facilitate the use of this work as a resource for OA researchers, a website (OATargets) was constructed to allow searching of the curated data, prioritisation of targets from the network algorithm and visualisation of PPI interactions between OA genes (figure 4A). The database provides an PPI network coloured by the inferred effect on the OA phenotype, enabling exploration of the signalling neighbourhood of a gene. For example, *EZH2* interacts with several other OA genes illustrating the concept of OA pathways (figure 4B). This database is linked to the existing resource SkeletalVis to provide integration with over 700 skeletal gene expression profiles. Experiments with differential expression of a selected gene can be identified to assess tissue localisation or to find transcriptional regulators of that gene (figure 4C). Again, using *EZH2* as an example, it is dysregulated in several post-traumatic gene expression datasets. The knowledge base is publicly available at http://skeletalvis.ncl.ac.uk/OATargets/.

## Discussion

We have curated two decades of OA research to identify the large number of genes studied in OA animal models and have produced a database for future research. The generally consistent results between heterogeneous OA models support the robustness of the findings from these models. Several genes have complementary evidence from genetic and exogenous modulations that make promising putative human drug targets for further study. For instance, cartilage specific knockout of *Sirt1* increases susceptibility to both ageing and surgically-induced OA, while pharmacological intra-articular activation of *Sirt1* protects against surgically-induced OA.^41 42^ Collection of these data also highlights several cases with discrepancies between studies, providing important cautionary lessons in interpreting these data. Knockout of *Mink1* showed protective effects in an ageing model, but detrimental effects in a surgical model, within the same publication, indicating different models can give divergent conclusions⁠.^43^ Several gene perturbations showed a phenotype in one model, but no effect in another suggesting that molecular regulation of OA is disease-phenotype-dependent, for example, knockout of *Tlr4* protects against high-fat diet induced OA, but not post-traumatic OA⁠.^44–46^

OA is a joint-wide disease, so a range of tissues are examined for phenotypes in the identified studies, but most of the genetic perturbations are global/systemic or cartilage specific. The cell types targeted and timing of interventions between acute exogenous and global genetic or inducible genetic modulation may be responsible for some of the observed differences in studies examining the same gene. *Rheb* overexpression is protective in articular cartilage, but *Rheb* knockout in macrophages is also protective, suggesting caution should be employed when interpreting global knockouts or systemic drug treatments⁠.^47 48^ Different cells are known to be targeted in *Col2-Cre* and tamoxifen-inducible *Col2-CreER* genetic modulations⁠.^49^ Furthermore, it is often unclear what cells are most affected by exogenous interventions⁠. Recent studies using surgical models reported inducible cartilage knockout of *Ezh2* to be detrimental, but treatment with an *Ezh2* inhibitor in the articular cavity was protective⁠.^50 51^ Drugs may have off-target effects and many studies do not assess if the drug at the selected dose was on target, which may contribute to results of a drug-based intervention against a designated target differing from an inducible genetic manipulation. These data suggest the need to look back at older results more critically, with the possibility of repeating gene modulations in other models.

Many genes have been studied in only one model, so it is unclear how generalisable results from such studies are. However, generally consistent findings were found between those genes that were studied in both spontaneous and surgical models, suggesting a core set of genes may be involved in both disease phenotypes. Subgroups of OA have previously been identified from cartilage genome-wide expression analysis of ‘end-stage disease’ (joint replacement) demonstrating the heterogeneity of the human disease⁠.^17^ We therefore suggest it is advisable to examine genes in multiple models of OA, and at multiple time points or stages of progression, potentially representing different subpopulations of human OA patients. Furthermore, where possible the use of tissue-specific genetic modification will enable a clearer understanding of the potential origin of OA phenotypes.

While bringing these studies together is useful for understanding OA pathways, combining the results from these variable studies has limitations. We present an inclusive list of findings using different scoring systems and variable statistical power to detect differences between conditions. Additionally, we do not record the sex of the animals studied, but the majority of DMM studies use only male mice. It is challenging to quantify the relative severity of the induced OA between studies due to differences in scoring systems which are usually semiquantitative and subjective. The OA models examined are heterogeneous, variations of surgical models have differences in OA severity and the severity induced within a given model may differ between surgeons.^10^ The approach of labelling genes as protective or detrimental is a simplification as genes may have a homeostatic role requiring calibrated expression for joint health or have a differential function during the early to late disease process. For example, either overexpression or knockout of *Ttr* in a surgical model is detrimental to joint function⁠.^52^ A more granular, tissue level annotation of the OA phenotypes would be interesting to explore in the future as gene modulations may vary in the tissues they affect. However, this is currently challenging to perform meaningfully given the above caveats and as most studies do not evaluate all individual tissue phenotypes. The idea of using human omics data to prioritise animal model research (ie, ‘the bedside-to-bench’ approach) is attractive as inclusion aids relevance to human disease and potential translatability. Future comparison to proteomics and protein activity data would add an important layer of evidence, particularly as the latter may correlate poorly with transcriptomics data due to post-translational regulation, that is, phosphorylation, or protease activation/inhibition.

OA is a polygenic disease and the network analysis suggests the close network proximity of many of the experimentally perturbed genes. Modulation of many individual genes can give rise to the same phenotype⁠.^40^ The observed network proximity in OA is likely influenced by both bias in publishing of tested genes by researchers, as well as the presence of disease pathways responsible for the OA phenotypes. Despite the bias in the data collected, the prediction of genes that are OA relevant allows inference of gaps in knowledge and prioritisation of research. The top ranked genes are potential candidates for future studies in animal or *in vitro* models. For example, the extracellular Wnt antagonist *WIF1* interacts with several Wnt proteins known to affect OA, is dysregulated in human OA transcriptomic datasets, has small-molecule tractability and has been reported to correlate with histological cartilage grade⁠.^53^ Understanding the redundancy and relatedness of genes within the same pathway in terms of OA phenotypes could be useful for reducing, essentially reiterative, animal model use. There is only limited negative data published and future access to such information would be very useful in better predicting genes that are likely to be functional. The current network prioritisation does not account for functional redundancy so is likely to include false positives, for example, *ADAMTS4* is highly ranked, but knockout in mice does not affect spontaneous or surgically induced OA.^54^

The next step of finding key drivers of the pathogenic processes that occur in human OA over a much longer time scale and that can be therapeutically targeted in humans to improve joint function at time points amenable to intervention is a major challenge. There is a prevalence of surgical models used in the exogenous studies. Interventional studies of the most promising targets, perhaps identified in post-traumatic OA models, in longer time course, ageing based models would be beneficial in understanding the impact of intervention timing and the long-term benefits of treatment. Target druggability and benefit-to-risk ratio for OA treatment must also be considered. We believe that providing a resource with multiple layers of evidence and tractability data will aid future work towards better OA target selection. For instance, evaluation of past clinical trial failures IL1 and TNF using OATargets shows they have mixed effects in the animal models, no genetic and limited transcriptomics support for their use in OA structural disease modification.^55^ Future inclusion of gene modulation effects on pain phenotypes would be useful for critical symptom-modifying drug target selection for OA.

This study has provided a resource for researchers to contextualise new data or explore existing publications. The OA genes can be used in tools to combine new differential expression datasets with the prior knowledge of OA joint damage-associated genes.^56^ ⁠This database provides researchers with means to mine new targets for evidence of interactions with known OA genes and examine cross-species and cross-model gene expression dysregulation. Ultimately, we hope ongoing addition to and use of the database will improve understanding of the molecular pathophysiology of OA joint damage and lead to the development of disease modifying therapies for this currently intractable condition.
