# Supplementary material for: OATargets: a knowledge base of genes associated with osteoarthritis joint damage in animals
Source: Ann Rheum Dis. 2020 Oct 19;80(3):376–83. doi: 10.1136/annrheumdis-2020-218344 (PMC7892386; doi:10.1136/annrheumdis-2020-218344)
Supplement: Supplementary data [file annrheumdis-2020-218344supp001.pdf]

Supplementary methods

Network expansion of OA associated genes

Network diffusion algorithms have been successfully used to predict genes involved in diseases using the principle that genes in close protein-protein interaction (PPI) network proximity to known disease genes may share biological functions and therefore be candidate disease modulators[1]. We used a network diffusion algorithm which models heat flow from animal model OA genes along interactions in a PPI network. The node scores at equilibrium are a measure of proximity to the known OA genes, since genes surrounded by OA genes will have a high heat flow. This method therefore allows systematic prioritisation of known OA genes by highlighting yet unstudied genes in similar pathways to the known OA genes. The OmnipathR (v1.0.0) and bioGRID (v3.5.186) collections of curated PPIs was downloaded and combined for use as a network.[2,3] The DiffuStats "raw" method was used to diffuse heat flow from the OA genes across the largest connected component of the network with default parameters and the genes in the network were subsequently ranked by the heat score.[4] For repeated cross-validation, 80% of known OA genes were randomly sampled 100 times and used as input for the network diffusion. The ranks of the remaining sets of 20% known OA genes compared to unlabelled genes were used to test the predictive performance of this approach. Newly reported animal model OA associations from the 2020 OARSI conference abstracts were used as a validation of predictive performance on new findings. The Wilcox test was used to test the expected and the observed protein ranks. GWAS signal variants were retrieved from a recent review.[5] Target drug tractability information was obtained from the OpenTargets platform.[6]

References

1 Barabasi A-L, Gulbahce N, Loscalzo J. Network medicine: a network-based approach to human disease. *Nat Rev Genet* 2011;**12**:56–68. doi:10.1038/nrg2918

2 Türei D, Korcsmáros T, Saez-Rodriguez J. OmniPath: Guidelines and gateway for literature-curated signaling pathway resources. Nat. Methods. 2016;**13**:966–7. doi:10.1038/nmeth.4077

3 Stark C. BioGRID: a general repository for interaction datasets. *Nucleic Acids Res* Published Online First: 2006. doi:10.1093/nar/gkj109

4 Picart-Armada S, Thompson WK, Buil A, *et al.* DiffuStats: An R package to compute diffusion-based scores on biological networks. *Bioinformatics* 2018;**34**:533–4. doi:10.1093/bioinformatics/btx632

5 Reynard LN, Barter MJ. Osteoarthritis year in review 2019: genetics, genomics and epigenetics. Osteoarthr. Cartil. 2020;**28**:275–84. doi:10.1016/j.joca.2019.11.010

6 Carvalho-Silva D, Pierleoni A, Pignatelli M, *et al.* Open Targets Platform: New developments and updates two years on. *Nucleic Acids Res* 2019;**47**:D1056–65. doi:10.1093/nar/gky1133
