# Supplementary material for: OATargets: a knowledge base of genes associated with osteoarthritis joint damage in animals
Source: Ann Rheum Dis. 2020 Oct 19;80(3):376–83. doi: 10.1136/annrheumdis-2020-218344 (PMC7892386; doi:10.1136/annrheumdis-2020-218344)
Supplement: Supplementary data [file annrheumdis-2020-218344supp004.pdf]

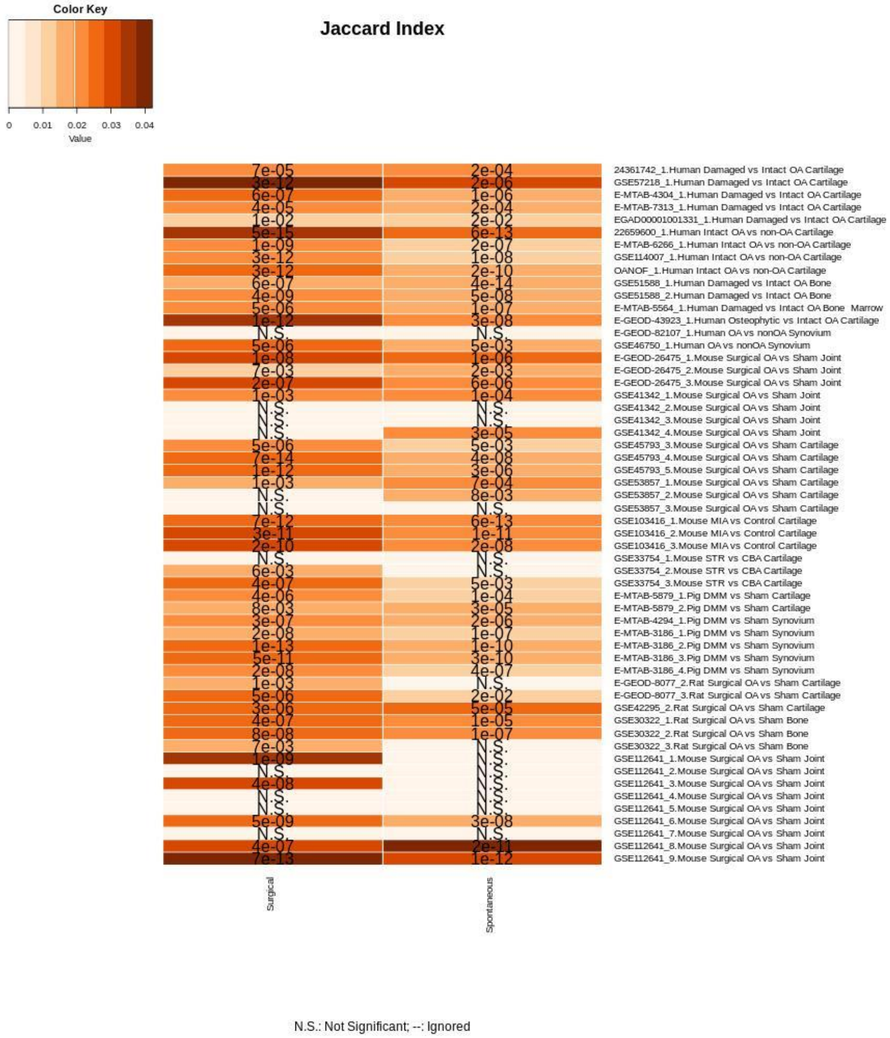

Supplementary Figure 1: Differential expression of genes studied in animal models by model

The Fishers exact test BH adjusted p-values for the overlap between gene expression datasets and OA genes studied in spontaneous or surgical models are shown. The cells are coloured by the Jaccard Index which shows the overlap relative to the number of differentially expressed genes.
